# Case Report: Clinical and Procedural Implications of Ommaya Reservoir Implantation in Cystic Brain Metastases Followed by Radiosurgery Treatment

**DOI:** 10.3389/fsurg.2022.901674

**Published:** 2022-05-16

**Authors:** Junhui Lv, Zhuoxuan Wu, Kun Wang, Yirong Wang, ShuXu Yang, Weidong Han

**Affiliations:** ^1^ Department of Neurosurgery, Sir Run Run Shaw Hospital, College of Medicine, Zhejiang University, Hangzhou, China; ^2^ Department of Medical Oncology, Sir Run Run Shaw Hospital, College of Medicine, Zhejiang University, Hangzhou, China

**Keywords:** cystic brain metastases, stereotactic cyst aspiration, Gamma Knife radiosurgery, Ommaya reservoir, craniotomy

## Abstract

**Background:**

Therapy for large or deep cystic brain metastases is a troublesome procedure in clinical departments. Stereotactic cyst aspiration, combined with Gamma Knife radiosurgery, can be an effective treatment for cystic brain metastases. However, there is still a possibility that a reaccumulation of cystic fluid may lead to poor efficacy or even reoperation.

**Case presentation:**

We present a case of a 67-year-old man who was diagnosed with lung cancer brain metastasis. The intracranial lesion seen on imaging appeared to be cystic and located deep inside the brain with associated limb dysfunction. The patient did not respond well to chemotherapy and underwent cyst aspiration with Ommaya reservoir implantation under neuronavigation. Repeated cystic fluid reaccumulation and exacerbation of symptoms occurred during treatment. We performed repeated aspiration via the Ommaya reservoir to control the symptoms and combined it with radiotherapy. During the follow-up period of 14 months, the intracranial tumor was effectively and satisfactorily controlled.

**Conclusions:**

We highlight that Ommaya reservoir implantation during stereotactic cyst aspiration is necessary to prevent fluid reaccumulation, thereby avoiding the need for a second surgical procedure.

## Introduction

Brain metastases are the most common central nervous system tumors and confer a grave prognosis to patients with a median survival of less than 1 year (1). Brain metastases can be radiographically cystic or solid (2). Patients with cystic metastases tend to have a worse prognosis than those with solid metastases (3). Generally, radiosurgery is less effective for patients with cystic metastases, especially for tumors >3 cm in diameter (4), and surgical resection is not only traumatic but also increases the risk of leptomeningeal dissemination (5). Another method (6) is stereotactic cyst aspiration (SCA), directly followed by Gamma Knife radiosurgery (GKRS). SCA can quickly reduce the volume of lesions, making it suitable for GKRS afterward. Our center does not have the necessary infrastructure to complete this one-step approach. In this article, we report a case treated with additional Ommaya reservoir (OR) insertion after SCA, followed by radiosurgery up to one and a half months later. Repeated cystic fluid reaccumulation and exacerbation of symptoms occurred during treatment. We performed repeated aspiration via the OR to help the patient cope with this situation and combined it with radiotherapy. We emphasize the advantage of OR placement, which is an effective tool for sufficient reduction in volume before and after radiosurgery in the case of fluid reaccumulation without reoperation.

## Case Presentation

**Figure 1:**
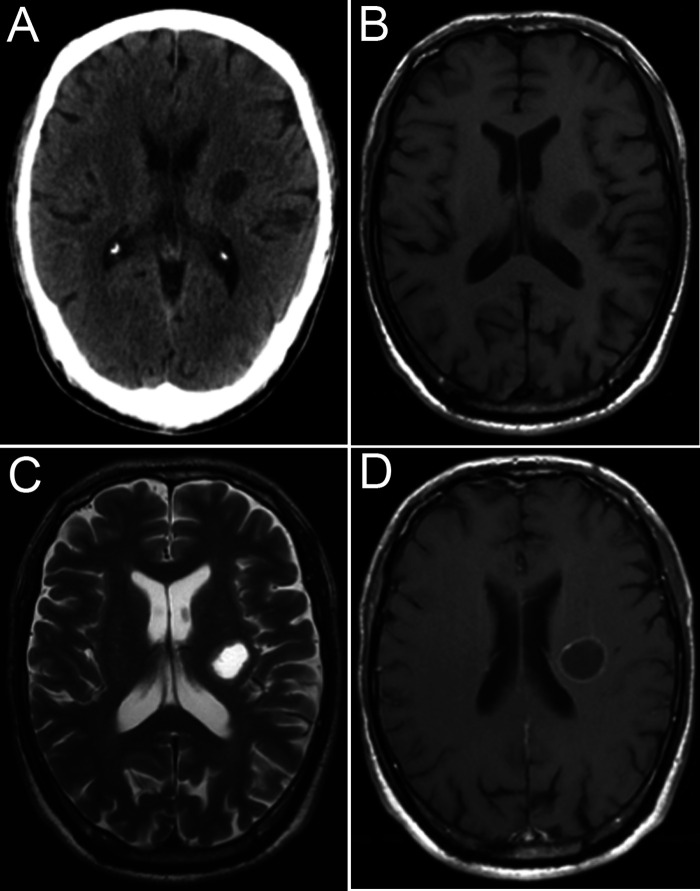
Image at admission. CT image (**A**), MR T1 image (**B**), MR T2 image (**C**), and MR T1-weighted image (**D**).

A 67-year-old man was admitted to the neurosurgery department of our hospital with a 1-month history of right upper limb weakness. He had no other medical history other than hypertension. Cranial computed tomography (Figure 1A) showed a circular focus of low density beside the left lateral ventricle. The lesion appeared hypointense on T1-weighted imaging and hyperintense on T2-weighted imaging, and rim enhancement was seen on postcontrast T1-weighted imaging (Figures 1B–D).

A further systemic evaluation revealed a pulmonary lesion, and transbronchial lung biopsy pathology indicated lung squamous cell carcinoma. The patient was transferred to the oncology department for chemotherapy treatment with paclitaxel plus carboplatin.

**Figure 2:**
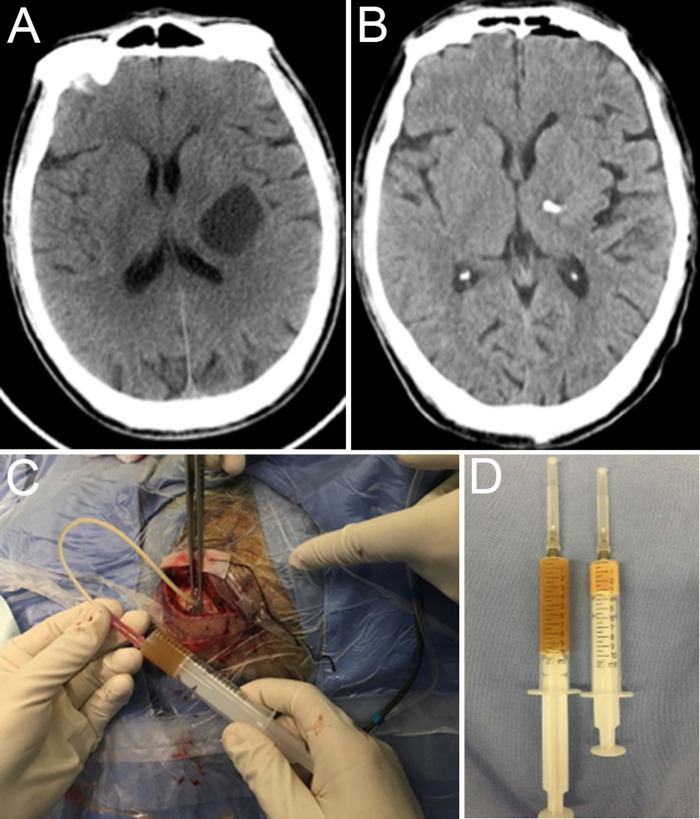
Preoperative CT reexamination (**A**), postoperative CT (**B**), intraoperative cystic fluid aspiration (**C**), and intraoperative specimen (**D**).

After two cycles of chemotherapy, his right muscle strength gradually decreased. Cranial computed tomography (Figure 2A) demonstrated that the intracranial cystic metastasis had progressed compared with the previous case.

**Figure 3:**
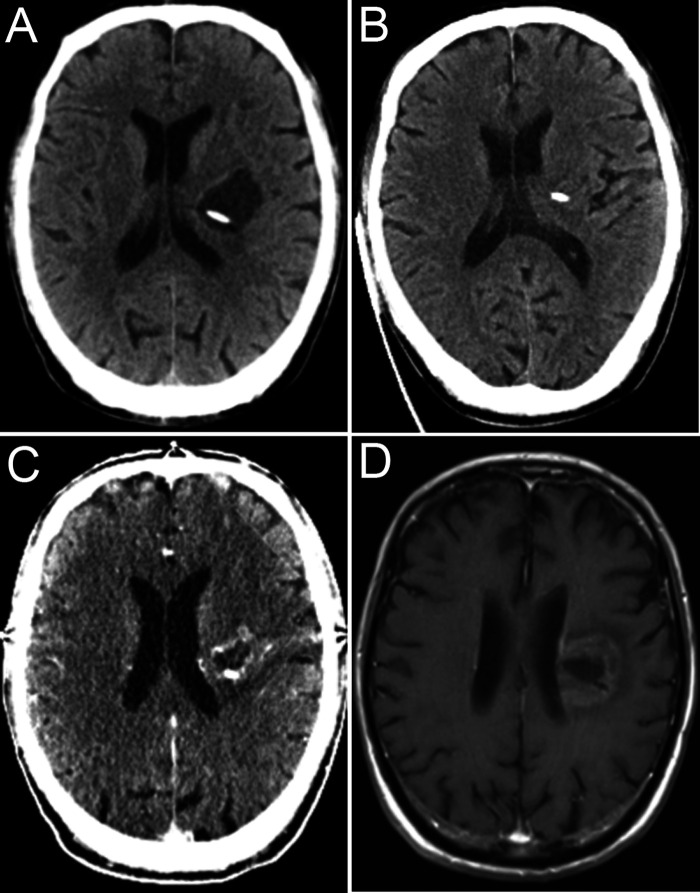
CT image showing fluid reaccumulation (**A**). CT image immediately after aspiration via the OR (**B**), CT image after first radiosurgery (**C**), and final imaging follow-up (**D**).

The patient underwent cyst aspiration (Figure 3C) and OR implantation under neuronavigation. A total of 13 ml of yellow viscous liquid (Figure 2D) was drawn during the operation. Postoperative computed tomography (Figure 2B) showed that the lesion had almost disappeared, and his right muscle strength had recovered.

The cyst immediately (Figure 3A) returned, with a progressive decline in contralateral limb muscle strength. We promptly implemented cystic fluid aspiration (Figure 3B), and the patient’s symptoms were relieved. Our hospital is not equipped to carry out Gamma Knife therapy. Therefore, we performed stereotactic body radiation therapy (SBRT) after aspiration to prevent a recurrence. Cystic lesions can still recur and become enlarged under such combined treatment. Repeated cystic fluid reaccumulation and exacerbation of symptoms occurred during treatment. We performed repeated aspirations via the OR to help the patient cope with such a situation. We performed SBRT again approximately half a year later (Figure 3C). However, we were unable to draw out the cyst fluid due to blockage of the OR before the last radiosurgery. Throughout the treatment, we received a total of five aspirations. The patient’s last radiographic follow-up was 14 months after onset (Figure 3D). The intracranial tumor was effectively and satisfactorily controlled. Due to the loss of follow-up, the patient’s current survival status is unknown.

## Discussion

Brain metastases often have associated cystic components. However, the mechanism of cyst formation has not been demonstrated yet. The breakdown of the blood–brain barrier is considered the possible cause (7), and the consensus is that cystic metastases tend to be more malignant than solid metastases (8). Therefore, cystic brain metastases usually progress rapidly and lead to serious clinical consequences.

A single large cystic brain metastasis is considered the operative indication, and surgical treatment can obviously relieve the clinical symptoms (9, 10). However, if the lesions are deeply seated or located in important functional areas, surgery can cause severe neurological dysfunction. Moreover, patients in poor general condition are intolerant to craniotomy operations. Stereotactic radiosurgery (SRS) is an alternative treatment option for brain metastases (8). However, cystic brain metastases are often too large to be suitable for SRS, and these types of tumors do not respond well to SRS (11). In this situation, we can use radioenhancers to increase the efficiency of SRS in the context of brain metastasis (12, 13) and reduce the cyst volume via SCA to make the lesion suitable for SRS (6).

Effective reduction of tumor volume is the key to subsequent treatment. The volumetric decrease reported in the literature ranged from 47.8% to 77.9% (6, 9, 14–17). Akito Oshima reported that the degree of tumor volume reduction depended on the location of the puncture target (16). He suggested that the tip of the puncture needle should be placed at the center of the cyst to ensure adequate volume reduction. Although the drainage tube was off-centered in our case, the cyst was close to disappearing. We believe that this is also related to the preponderance of cystic fluid, a thin cystic wall, and less tumor parenchyma.

SCA is a relatively safe procedure, and related complications are rare. Its complications include infection, hemorrhaging, neurological symptoms, and seizures, similar to stereotactic biopsy. One study reported that the SCA-related complication rate was 5.8%, and the mortality rate was 3.8% (6). The article attributed the higher mortality rate to the fact that the study population was all ASA IV. Another theoretical complication after SCA is cancer cells spreading through the aspiration needle tract. A few such complications have been reported. However, the literature reports only a 9% risk of needle biopsies leading to tumor cell seeding (18).

It is debatable whether additional ORs are inserted after SCA. Some studies have suggested that SCA without Ommaya insertion and directly followed by Gamma Knife therapy is an effective and time-efficient treatment (6, 14). However, there are many limitations to this one-step approach. First, most centers do not have the necessary infrastructure for a one-step approach to be a regular process. In our hospital, surgery and SRS are performed in two different departments. It is not easy to guarantee that Gamma Knife therapy can be performed immediately after SCA. If the interval between the two procedures is too long, the risk of fluid reaccumulation will increase. Second, the reaccumulation of cystic fluid can still occur after aspiration and GKRS. The local control rates ranged from 54.2% to 91.3% (6, 9, 15, 17, 19). OR insertion during SCA can be an additional tool for subcutaneous cyst aspiration in the case of fluid reaccumulation. In our case, the cystic fluid accumulated repeatedly. Although the fluid type was turbid (Figure 2D), we could still take subcutaneous cyst aspiration via the OR multiple times and avoided reoperation. In my opinion, the OR should be inserted to reduce the chances of repeated SCA.

## Conclusion

In our case, we selected SCA, combined with radiosurgery, which is less invasive than resection, to treat a deeply located cystic lesion. Although the cystic fluid was found to be turbid during surgery, we still implanted an OR during SCA. Because of the implanted OR, we solved the problem of cystic fluid reaccumulation in subsequent treatment.

We believe that OR implantation during cyst aspiration is necessary to prevent fluid reaccumulation, avoiding the need for a second surgical procedure. Although turbid cystic fluid may lead to tube blockage, OR implantation is still worth giving a try.

## Data Availability

The raw data supporting the conclusions of this article will be made available by the authors, without undue reservation.
